# Effects of immune exhaustion and senescence of innate immunity in autoimmune disorders

**DOI:** 10.1590/1414-431X2024e13225

**Published:** 2024-06-17

**Authors:** A.L.S. Cunha, S.F. Perazzio

**Affiliations:** 1 Divisão de Reumatologia, Universidade Federal de São Paulo, São Paulo, SP, Brasil; 2 Divisão de Imunologia, Laboratório Fleury, São Paulo, SP, Brasil; 3 Laboratório Central, Hospital das Clínicas, Faculdade de Medicina, Universidade de São Paulo, São Paulo, SP, Brasil

**Keywords:** Immune exhaustion, Immune senescence, Innate immune system, Autoimmunity and immunology

## Abstract

Innate immune system activation is crucial in the inflammatory response, but uncontrolled activation can lead to autoimmune diseases. Cellular exhaustion and senescence are two processes that contribute to innate immune tolerance breakdown. Exhausted immune cells are unable to respond adequately to specific antigens or stimuli, while senescent cells have impaired DNA replication and metabolic changes. These processes can impair immune system function and disrupt homeostasis, leading to the emergence of autoimmunity. However, the influence of innate immune exhaustion and senescence on autoimmune disorders is not well understood. This review aims to describe the current findings on the role of innate immune exhaustion and senescence in autoimmunity, focusing on the cellular and molecular changes involved in each process. Specifically, the article explores the markers and pathways associated with immune exhaustion, such as PD-1 and TIM-3, and senescence, including Β-galactosidase (β-GAL), lamin B1, and p16^ink4a^, and their impact on autoimmune diseases, namely type 1 diabetes, rheumatoid arthritis, systemic lupus erythematosus, and immune-mediated myopathies. Understanding the mechanisms underlying innate immune exhaustion and senescence in autoimmunity may provide insights for the development of novel therapeutic strategies.

## Introduction

Innate immunity is involved in inflammatory response but its uncontrolled activation has been somehow linked to autoimmunity. As in any other inflammatory pathway, each innate component is tightly modulated by other redundant proteins. Therefore, defects in any of these checkpoints may cause a spectrum of immunodeficiency and autoinflammatory or autoimmune diseases (1).

Cellular exhaustion and senescence may also contribute to innate immune tolerance breakdown. Immune cells become exhausted when unable to adequately respond after a challenge with specific antigens or stimuli (2). On the other hand, cell senescence is defined by its inability to replicate as telomeres reach a critical length or DNA is irreparably damaged (3). Although exhaustion and senescence may be considered part of the physiological cell maturation, they may also impair overall immune system function and destabilize homeostasis over time, finally culminating with self-tolerance breakdown and the emergence of autoimmunity (4).

The innate immune system is particularly affected by immune exhaustion and senescence, which can be observed by the constant turnover of effector cells and soluble factors. Nevertheless, to the best of our knowledge few articles address the influence of innate immune exhaustion and senescence on autoimmune disorders. Herein, we aimed to describe the current findings of innate immune exhaustion and senescence on autoimmunity, mainly focusing on structural cellular modification and molecular pathways involved in each process.

### Immune exhaustion

Cell surface costimulatory signaling modulators are hallmarks of immune exhaustion [Table 1, (5-7)] and can be easily assessed by using different tools like flow cytometry, immunohistochemistry, and western blot. Programmed cell death protein 1 (PD1) and its ligands 1 (PD-L1) and 2 (PD-L2) constitute an important regulatory pathway that impedes costimulatory signaling during T cell activation (8). Simultaneous co-expression of PD1/PDL-1 with other inhibitory receptors on T cells, such as lymphocyte activation gene 3 protein (LAG3), 2B4/CD244, CD160, T cell immunoglobulin domain and mucin domain-containing protein 3 (TIM3), and cytotoxic T-lymphocyte-associated protein 4 (CTLA4) is highly suggestive of an immune exhaustion phenotype. A PD-1/PD-L1 pathway neutralization-induced bystander effect on NK cells was observed in an experimental IL-2-dependent exhaustion mouse model, resulting from the global competition that exists between NK and CD8^+^ T cells for IL-2 as a key regulator of these cells' activation (9). PD-1 and TIM-3 combined target may be the most efficacious manner to improve anti-tumor response *in vivo* as demonstrated in BALB/c and C57BL/6 mice models (10). Interestingly, the higher the number of inhibitory receptors expressed by exhausted T cells, the more severe the exhaustion process evolves, a pattern apparently also shared by innate immune cells. Lin et al. (11) showed that LPS stimulus caused neutrophil exhaustion after TIR domain-containing adapter molecule 2 (TICAM2) and PD1-mediated activation of Src family kinases (SFK). PD1/PD-L1 are also expressed on exhausted murine monocytes/macrophages and dendritic cells (DC) from septic peritonitis induced by a cecal ligation murine model, similarly to those derived from septic shock patients (12). Hence, as PD1/PD-L1 and CTLA-4 are currently important targets for cancer immunotherapy (13), other exhaustion markers, such as TIM3, have similarly been considered (14).

**Table 1:** Key cell surface costimulatory signaling modulators associated with innate immunity exhaustion.

Innate cells	Exhaustion markers	Reference
DC	PD1	5
Macrophages	PD1	5
	TICAM2	14
Mast Cells	PD1 and TIM3	6
Neutrophils	PD1 and PD-L1	11
	TICAM2	11
	ICAM1, CD11b	7

DC: dendritic cells; PD1: programmed cell death protein 1; PD-L1: programmed cell death protein ligand 1; TICAM2: TIR domain-containing adapter molecule 2; TIM3: T cell immunoglobulin domain and mucin domain-containing protein 3; ICAM1: intercellular adhesion molecule-1; CD11b: integrin alpha-M.

### Immune senescence

Cells become senescent when their DNA replication ability progressively deteriorates, resulting in striking metabolic modifications and expression of immune senescence markers. There is compelling evidence that immune senescence plays a significant role in immune dysfunction and disability in older people (15). Elderly have worse T cell memory responses than young people, which may likely be the result of a combination of factors including reduced TCR repertoire diversity, poor T cell assistance, and substantial decreased naive T cell count along with aging, as shown in HIV-infected elders (16). Previous studies indicate that the ageing process or repeated cell activation cycles significantly impede the ability of immune cells to start primary responses against novel antigens, although immune responses against previously recognized antigens may still be conserved (17). This difficulty is usually increased by an impairment of innate immune effector cells, mainly neutrophils and monocytes, and may result in susceptibility to infectious diseases (18). The microbicidal function of senescent neutrophils is highly impaired, mostly because of a reduced chemotactic ability, which in turn delays tissue recovery as shown in mouse lungs (19). Moreover, reduced neutrophil phagocytic activity against opsonized *E. Coli* and Fcγ receptor CD16 surface expression were previously shown in elderly humans (20).

Impaired intracellular signaling have also been reported in senescent neutrophils, including reduced calcium intake, decreased kinase and phosphatase activities [namely, phosphoinositide-3 kinase (PI-3K), mitogen-activated protein kinase (MAPK), protein kinase B, and Src homology region 2 domain-containing phosphatase-1 (SHP-1)], and impaired Janus kinase (JAK)-signal transducer and activator of transcription (STAT) interaction (21). Altered intracellular signaling in senescent neutrophils may also hamper oxidative burst and phagocytic activity. For most other aspects of neutrophil senescence our understanding is still incomplete.

**Figure 1:**
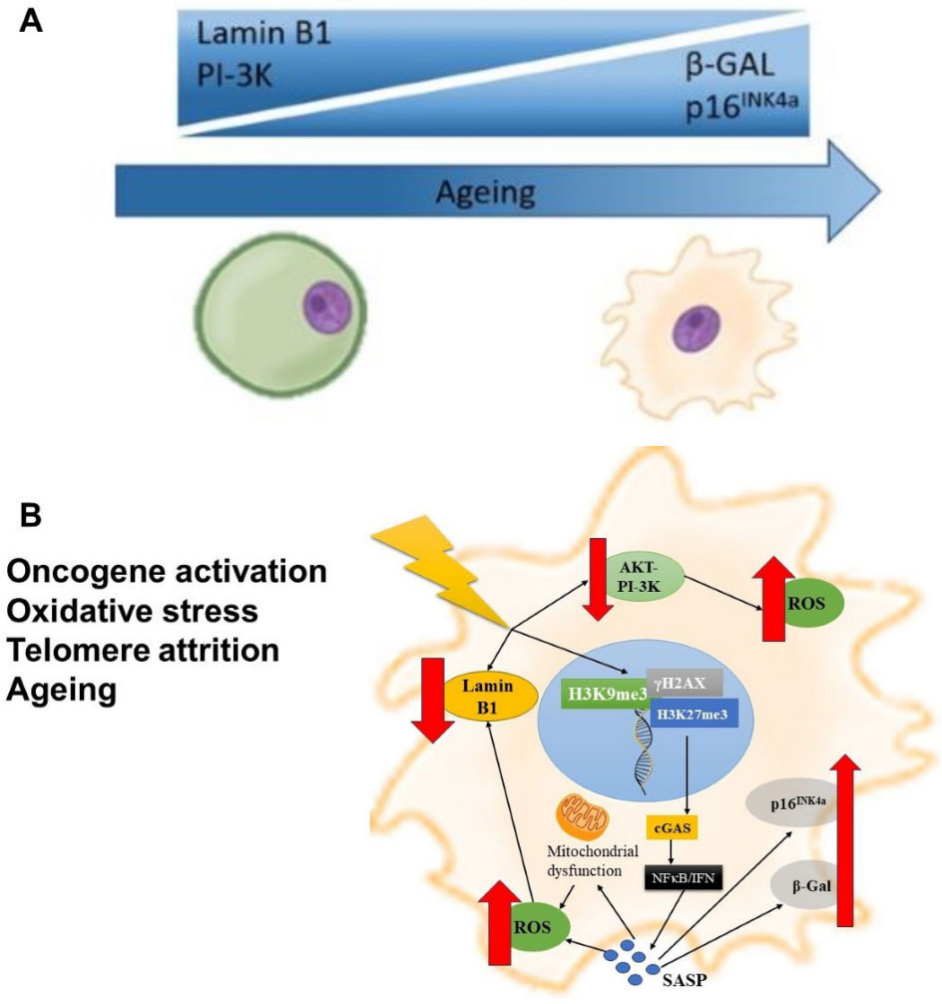
**A**, Main variations in senescence intracellular markers with ageing. **B**, The senescent stage: innate sensing involves multiple stressors, such as telomere attrition, oxidative stress, irradiation, ageing, and oncogene activation. Any stressor may induce three main senescence responses: i) AKT-dependent phosphoinositide 3-kinase (PI-3K) downregulation, which triggers reactive oxygen species (ROS) production, causing DNA damage; ii) direct double-stranded DNA damage releasing fragments enriched with yH2AX and repressive histone markers (H3K9me3, H3K27me3); and iii) lamin B1 downregulation. Upon disruption of the nuclear envelope favored by loss of lamin B1, yH2AX-, H3K9me3-, and H3K27me3-enriched DNA fragments are recognized by cyclic GMP-AMP synthase (cGAS), which activates nuclear factor κB (NF-κB) and interferon (IFN) pathways, culminating with senescence-associated secretory phenotype (SASP). SASP reinforces and amplifies senescence in a paracrine manner, activating immune cells for senescence immune surveillance, which in turn increases mitochondrial dysfunctional ROS release and upregulates β-galactosidase (β-GAL) and p16^INK4a^. Moreover, ROS overproduction is a major lamin B1 downregulator, feeding back the whole senescent process.

Other features of immune cell senescence have also been described, mainly leading to the irreversible pause of cell growth and development of a proinflammatory senescence-associated secretory phenotype (SASP; Figure 1 and Table 2) (22) such as β-galactosidase (β-GAL) and p16^INK4a^ (23). As a component of the cyclin-dependent kinase (CDK) inhibitors family, p16^INK4a^ blocks retinoblastoma protein, ultimately impeding S-phase entry and cell growth (24). Liu et al. (25) demonstrated a senescent phenotype in murine peritoneal macrophages derived from hybrid C57BL6/129SvEv transgenic model. Clearance of p16^INK4a^-expressing cells attenuates senescence and improves the healthy lifespan of a progeroid mouse model and aged control mice, as β-GAL was upregulated after p16^INK4a^ activation (25).

**Table 2:** Physiologic features of the main components involved in senescence-associated secretory phenotype (SASP).

SASP component	Physiology	Reference
Phosphoinositide-3 kinase (PI-3K)	Regulates cell proliferation, adhesion, survival, and motility.	21
Mitogen-activated protein kinase (MAPK)	Regulates cell proliferation, responses against stress factors, apoptosis, and immune defense.	21
Protein kinase B (PKB)	Regulates glucose metabolism, apoptosis, cell proliferation, transcription, and migration.	21
Src homology region 2 domain-containing phosphatase-1 (SHP-1)	Regulates cytokine signal transduction and modulates cell proliferation, differentiation, survival, and apoptosis.	21
Janus kinase (JAK)	Involved in cell growth, survival, maturation, and differentiation in a variety of cell lineages, especially immune cells.	21
Activator of transcription (STAT)	Regulates cell growth, survival, and differentiation.	21
Β-galactosidase (β-GAL)	Cleaves terminal β-d-galactose residues, such as lactose, keratin sulfates, and sphingolipids.	23,25
p16^ink4a^	Regulates cell cycle arrest.	23-25
Lamin B1	Provides stability of intermediate filaments in cytoskeleton and is a scaffolding component of the nuclear envelope.	26-28

Another SASP biomarker is lamin B1, a structural cell nuclear component involved in regulating many nuclear functions (26). Lamin B1 is downregulated in ultraviolet radiation (UV) *in vitro*-induced human senescent cells; similar evidence was demonstrated upon chronic *in vivo* UV exposure and skin regeneration (27). In addition, lamin B1 gene and protein expression declined in UV-induced murine senescence (28).

The cell maturation process is also affected by immune senescence, as human monocytes and dendritic cells (DC) progressively decline with time. Paradoxically, while senescent monocyte absolute count increases with ageing, peripheral macrophage number decreases and they become progressively resistant to Toll-like receptor (TLR) activation (29). Bella et al. (30) demonstrated that LPS-stimulated interleukin (IL)-12 and IL-10 *in vitro* production by murine senescent monocytes and DC is impaired. On the other hand, IL-6, IL-8, and IL-1α production increases as cells become senescent. IL-1α blockade in senescent cells markedly reduced IL-6 and IL-8 secretion (31). Similar dysfunction was demonstrated in senescent neutrophils and monocytes, whose TLR2/6, 3, 5, and 9-stimulated cytokines *in vitro* production is also totally defective (21). Moreover, a previous study showed impairment of TLR gene expression in C57BL/6 mice splenic and peritoneal senescent macrophages (32).

## How exhausted or senescent innate effector cells may affect autoimmunity pathophysiology

Cell exhaustion or senescence represents a state of cellular dysfunction characterized by suppressed cellular functionality. Within the context of autoimmunity, exhausted or senescent cells exhibit impaired functionality, thereby compromising immune system capacity of effectively eliminating pathogens, neoplasms, and autoreactive cells (33). Experimental evidence has demonstrated the presence of exhausted and senescent cells in both human and murine systems, highlighting their involvement in autoimmunity.

### Type 1 diabetes (T1D)

Diana et al. (34) showed that pancreatic beta cell death increases tissue migration and activation of B lymphocytes, neutrophils, macrophages, and plasmacytoid DC in young female non-obese diabetic (NOD) mice. Neutrophils cultivated *in vitro* in highly concentrated glucose medium developed SASP, however, it is still unclear whether glucose-induced senescence impairs neutrophil extracellular traps (NET) release (35), oxidative burst (36), and phagocytic activity (37). Although neutrophil exhaustion may impede T1D progression, it would also increase the risk of infectious diseases, as observed in diabetic patients (38).

Hyperglycemia-induced SASP amplifies diabetes-related endovascular and tissue inflammation and insulin resistance, and inhibits extracellular matrix production, thus creating a vicious circle. T1D SASP induces proinflammatory M1 macrophages maturation via NF-κB activation and increases reactive oxygen species (ROS) production and intracellular acidosis. Furthermore, histopathological analysis of diabetic wounds shows a protracted population of M1 phenotype-polarized senescent macrophages and a low expression of NLRP3, caspase1, and IL-1 (39). Interestingly, transcriptome of M1 phenotype hyperglycemic medium-induced THP-1 cells revealed a clear SASP-like signature, as IL-1α, IL-6, IL-8, PAI-1, TGF-β, TNF-α, MCP-1, ICAM-1, and IGFBP6 gene expression were strikingly upregulated (40). The histopathology analysis of diabetic mouse incisional wounds revealed a chronic inflammatory infiltrate enriched with senescent C-X-C motif chemokine receptor 2 (CXCR2)-positive macrophages (41). CXCR2 is a pro-fibrotic inflammatory chemokine receptor associated with SASP in primary human dermal fibroblasts (42). Immune senescence is a primary determinant of diabetic wound healing failure and closely linked to diabetic complications, which are a major cause of morbidity and shortened lifespan (40).

## Rheumatoid arthritis (RA)

Monocytes and neutrophils have emerged as key players in synovial inflammation and cartilage damage. Senescent monocytes have gained attention due to their shorter telomere length and mainly develop a non-classical (CD14^dim^CD16^bright^) pro-inflammatory phenotype (43). Notably, senescent non-classical monocytes express chemokine receptors that facilitate their migration to inflamed tissues and senescence-associated β-galactosidase (44).

Neutrophils, on the other hand, may play a crucial role in inducing a senescent phenotype in neighboring cells, including monocytes. As their involvement in cartilage damage and destruction is attributed to the release of ROS (45) and telomeres are particularly sensitive to oxidative stress, ROS release by RA neutrophils may induce neighboring cell senescence by significant telomere shortening (46). When co-cultured with neutrophils, human fibroblasts rapidly express senescence markers and shorter telomeres compared with control fibroblasts cultured alone. Moreover, the rate of dysfunctional telomeres, characterized by their association with DNA damage response factors (namely, telomere-associated foci, p16^Ink4a^, and p21) were increased in neutrophil-induced senescent cells. Noteworthy, premature senescence and telomere damage were prevented when extracellular ROS were scavenged by adding recombinant catalase (47).

## Systemic lupus erythematosus (SLE)

Similar to RA, neutrophil dysregulation also contributes to SLE pathogenesis. NET release probably exposes DNA and encrypted nuclear proteins to the immune system in SLE, culminating with autoantibody production, such as anti-double stranded DNA and anti-acetylated/methylated histones (45).

SLE neutrophils usually peak faster and produce higher ROS levels than those from healthy individuals (48). Nevertheless, neutrophils from active SLE patients paradoxically produce lower ROS levels than those from individuals with inactive SLE, probably due to neutrophil exhaustion (49). Conversely, peripheral PD-L1-expressing neutrophil count of SLE patients with active or severe disease is higher than those with inactive and milder conditions (50).

Low-density granulocytes (LDG), a specific subset of neutrophils, have emerged as a captivating area of investigation in the field of SLE. LDG rely on the lower density compared to conventional neutrophils, with isolation typically achieved by density gradient centrifugation techniques (51). LDG can exhibit an enhanced pro-inflammatory profile, characterized by heightened cytokine synthesis including TNF-α, IL-6, IL-8, and IFN (52) and are commonly elevated in peripheral blood of active lupus patients, especially those presenting vasculitis, cutaneous manifestations, or high anti-double stranded DNA titers (52). Additionally, LDG display an increased propensity for spontaneous production of NET (53), further contributing to the pathogenesis of SLE when taken together (51). As neutrophil hyperactivation induces degranulation and NET release, thus reducing cell density and resulting in exhaustion, one can hypothesize that LDG may play a role in neutrophil senescence. However, further comprehensive investigations are warranted to fully understand the role of LDG in SLE pathogenesis, particularly regarding their impact on senescence or exhaustion profiles in innate immune cells (54).

## Immune-mediated necrotizing myopathy (IMNM)

IMNM is a specific form of autoimmune myopathy distinguished by pronounced weakness in the proximal muscles, myofiber necrosis, and infiltration of inflammatory cells as neutrophils and macrophages (55). Knauss et al. (56) showed the high expression of PD-1, LAG-3, and TIM-3, a classic T cell exhausted phenotype, in muscle specimens extracted from 12 IMNM patients. Moreover, the authors also detected high expression of PD-L1 on macrophages and PD-L2 on myofibers. Interestingly, PD-L2 staining in myofibers was partially overlapping PD1 staining on CD3^+^ T lymphocytes, implicating the formation of the so called “immunologic synapses” and a potential role of PD-L2/PD1 interaction in modulating T-cell activation and macrophages cells.

PD-1 contributes to skeletal muscle regeneration by facilitating the transition of macrophages from a proinflammatory to an anti-inflammatory phenotype (57). IFN-γ, on the other hand, stimulates the formation of proinflammatory macrophages, which seem to impede myogenesis *in vitro* (57). However, Zhuang et al. (57) surprisingly revealed that blocking IFN-γ signaling of PD-1 knockout models actually exacerbated inflammation in the injured muscle, impeded muscle regeneration, and intensified muscle fibrosis. This detrimental effect was attributed to the inhibition of macrophage infiltration and transition from a proinflammatory to an anti-inflammatory state, associated with an increased influx of neutrophils into the muscle tissue.

Taken together, all these pieces of evidence support the hypothesis that anti-PD-L1 therapy holds the potential to ameliorate inflammation in IMNM. However, this hypothesis is currently being investigated, and preliminary data thus far have yielded discouraging results. Recent advancements in the field have led to a growing utilization of immune checkpoint inhibitors (ICI) across a wide range of malignancies. Nevertheless, this therapeutic strategy has unveiled a novel spectrum of adverse effects, especially in myositis induced by ICI therapy, which has an high mortality rate when co-occurring with other autoimmune manifestations such as myocarditis and myasthenia gravis (58).

## Multiple sclerosis

Multiple sclerosis (MS) is the most common chronic inflammatory, demyelinating, and neurodegenerative disease of the central nervous system in young adults (59). The global population of individuals over 65 years old with MS is on the rise as the life expectancy for those living with MS has improved (60). With this growing awareness, the challenges associated with aging, immunosenescence, and MS are also being recognized. These challenges also include a limited understanding of the long-term effects of disease-modifying therapies.

Two distinguished phases of MS's pathophysiology are recognized: early inflammatory and progressive phases (61). While during the first phase, the blood-brain barrier (BBB) is disrupted, allowing peripheral adaptive immune cell infiltration into the central nervous system (CNS), during the progressive phase, T and B cells influx is reduced as the BBB is closed and the inflammation is sustained by innate CNS-resident microglia and astrocytes. These cells produce cytokines, such as TNF-α and IL-6, and release ROS, culminating with myelin damage. While microglia and astrocytes become primed into a pro-inflammatory phenotype, their phagocytic activity is reduced and they progressively acquire a clear SASP. Improper clearance of myelin debris occurs, and oligodendrocyte progenitor cell recruitment and differentiation become less effective (62). These successive events become self-sustained and are amplified by senescent processes, resulting in a significant oxidative burst that leads to mitochondrial DNA damage-induced dysfunction, energy failure, and axonal loss. Moreover, cell cycle arrest and phenotypic changes in senescent cells might affect their functions and their regenerative capacity (63).

## Immune exhaustion and senescence-blocking factors of innate effector components

Modern lifestyle with a high-fat diet and excessive alcohol consumption, obesity, sedentary lifestyle, and smoking are important causes of low-grade chronic systemic inflammation, which puts the immune homeostasis in a state called inflammaging (64,65). Obesity, hyperglycemia (66), and sedentary lifestyle (67) increase proinflammatory cytokine production, such as IL-6 and TNF-α, and shift the memory:naive T cell ratio towards mature forms in humans (68). A similar immunophenotype has been described in peripheral T cells of mice and other chordates (69). Fat deposits stimulate neutrophil (70,71), macrophage, and T cell recruitment into the adipose tissue, which may dysregulate immune response and accelerate immune senescence and exhaustion (72).

To investigate the association between obesity and senescent cell accumulation, Ogrodnik et al. (73) studied the role of senescence in obesity-related neuropsychiatric disorders of the INK-ATTAC mouse model, which allows p16^Ink4a^-expressing cell elimination. The researchers found that obesity-induced senescent glial cells in the vicinity of the lateral ventricle, a region associated with adult neurogenesis, exhibited excessive fat deposits. Interestingly, neurogenesis was restored by clearing out senescent cells from leptin knockout mice fed with a high-fat diet.

In addition, adipocyte hyperplasia and hypertrophy increase adipocyte hypoxia, fatty acid metabolic dysregulation, chemokine secretion, adipocyte cell death, and inflammatory cell recruitment (74), ultimately inducing inflammaging and cell SASP, and generating a positive feedback loop that contributes to local and systemic inflammation. Studies demonstrated that both obese mice and human adipose tissues recruit pathogenic autoantibodies-secreting B cells (75) and favor proinflammatory cytokine secretion during aging, generating SASP senescent cells (76). Furthermore, a high-fat diet also upregulated p16^INK4a^ in cortical and hippocampal mouse neurons (77) and hepatocytes (78). Interestingly, possible functional impairments in adipose tissue neutrophils induced by aging are still unclear.

Sedentarism also induces inflammaging (79), but may be reverted by moderate physical activity (80). Recently, a 10-week program of regular walking increased neutrophil phagocytic and chemotactic activity after bacterial stimuli in elderly adults with rheumatoid arthritis (81). Moreover, neutrophil chemotactic activity of healthy elderly individuals who walked at least 10,000 steps per day was higher than that of age-matched adults who walked only 5000 steps per day (82). Similarly, corrective lifestyle interventions that prevent sedentarism and improve diet quality have the potential to prevent obesity, inflammation, aging, and the exhaustion process (72). Regular moderate-intensity physical activity suppresses IL-6, TNFα (83), and IL-1β (84), increases telomere length, and downregulates p16^INK4a^ (85), thus attenuating the hallmarks of aging.

## New approaches for anti-senescence therapy

Two distinct therapies targeting senescence have been identified: senolytic agents, a promising experimental class of drugs that selectively induce senescent cells to undergo apoptosis, like navitoclax, dasatinib plus quercetin, 17-DMAG (17-dimethylaminoethylamino-17-demethoxygeldanamycin), and senostatic agents, like ruxolitinib, rapamycin, and metformin, which inhibit SASP signaling pathways (86). On the other hand, probiotic bacteria in humans seem to present beneficial effects as anti-senescence therapy (87). A 4-week high-fiber diet with 5% inulin program suppressed *IL-1β*, *TNFα*, *IL-6*, *NLRP3*, and *TLR4* gene expression, induced *IL1RN* anti-inflammatory microglial gene expression, improved aging-associated neuroinflammation, and altered microbiome by reducing *Ruminococcus* spp and *Rikenellaceae* spp in adult and aged BALB/C mice (88). However, more studies in the area are still needed, focusing especially on anti-innate immunity senescence treatment.

## New approaches for anti-exhaustion therapy

Checkpoint inhibitors emerged as a transformative anti-tumor therapeutic strategy in oncologic patients by facilitating adaptive immunity activation but have also been considered an anti-exhaustion alternative therapy lately. A mouse model of cancer with B16F10 cell transplant demonstrated SASP with PD-1, TIM3, and LAG3 overexpression on CD8^+^ and CD4^+^ T cells, which were reversed after PD1 blockade (89). Anti-TIM3 also reversed SASP from a cecal ligation mouse model of sepsis by upregulating TLR4-induced NF-κB pathway activation in LPS-stimulated peritoneal macrophages (90). Certainly, additional studies are still needed to determine whether these agents could be critical in cell recovery.

## Conclusion

Here we have briefly described the current state of the research on immune senescence and exhaustion, uncovering the main pathways affecting innate immunity in the context of autoimmune diseases. We highlighted key SASP-induced components such as PD1, TICAM2, TIM3, Β-galactosidase (β-GAL), p16^ink4a^, and lamin B. NF-κB and IFN pathways also play a pivotal, though intricate, role in driving cell SASP. In addition, we discussed possible targeted interventions able to block immune senescence (senolytic agents like navitoclax, dasatinib plus quercetin, and 17-DMAG; and senostatic agents like ruxolitinib, rapamycin, and metformin) and exhaustion (especially checkpoint inhibitors). Innate immunity is our first line of defense and is mainly composed by short-lived cells, which may pose challenges in studying exhaustion and senescence due to their gradual nature, but the field holds significant untapped potential. Much remains to be explored in this domain, and it is evident that further studies are imperative to unravel the pathophysiological intricacies associated with these molecules and pathways. These endeavors may in turn contribute to the identification of novel therapeutic targets and improve our understanding of autoimmune diseases.
